# The Male Reproductive System of the Kissing Bug, *Rhodnius prolixus* Stål, 1859 (Hemiptera: Reduviidae: Triatominae): Arrangements of the Muscles and the Myoactivity of the Selected Neuropeptides

**DOI:** 10.3390/insects14040324

**Published:** 2023-03-28

**Authors:** Angela B. Lange, Anika Kisana, Jimena Leyria, Ian Orchard

**Affiliations:** Department of Biology, University of Toronto Mississauga, Mississauga, ON L5L 1C6, Canadaian.orchard@utoronto.ca (I.O.)

**Keywords:** accessory glands, seminal vesicle, testis, ejaculatory duct, proctolin, FMRFamide-like peptides, myosuppressin

## Abstract

**Simple Summary:**

The kissing bug, *Rhodnius prolixus*, is a blood-gorging insect that is medically important, being a principal vector of *Trypanosoma cruzi*, which causes Chagas disease. Understanding the reproductive biology of *R. prolixus* is, therefore, of some importance from a scientific perspective, but also in the medical context in order to control the spread of the disease. Here we show that the male reproductive system of *R. prolixus* is composed of muscular tissues performing contractions that aid in the transfer of sperm and other fluids into the female during mating. These contractions are further shown to be controlled by neuropeptides released by the nervous system that act on the receptors to either increase or inhibit the contractions. The male reproductive structures are, therefore, coordinated for successful copulation, whereby the transfer of sperm and fluid occurring during copulation is facilitated through a coordination of the contraction of the muscles within the male reproductive system. This study is critically important to provide novel options for pest management.

**Abstract:**

The gross anatomy of the male reproductive structures and their associated musculature are described in the blood-gorging vector of Chagas disease, *Rhodnius prolixus*. The male reproductive system is composed of muscular tissues each performing contractions that aid in the movement of sperm out of the testis into the vas deferens, seminal vesicle and then into the ejaculatory duct, along with proteins and lipids from the transparent and opaque accessory glands. Phalloidin staining shows the various patterns of muscle fiber layers, from thin circular to more complex crisscross patterns, implying subtle differences in the form of the contractions and movement of each of the structures, allowing for waves of contractions or twisting patterns. The transcripts for the receptors for proctolin, myosuppressin (RhoprMS) and for the extended FMRFamides are expressed in the various regions of the reproductive system, and the nerve processes extending over the reproductive structures are positive for FMRFamide-like immunoreactivity, as are neurosecretory cells lying on the nerves. Proctolin and AKDNFIRFamide are strong stimulators for the frequency of the contractions, and RhoprMS can inhibit the proctolin-induced contractions. Taken together, this work implicates these two families of peptides in coordinating the male reproductive structures for the successful transfer of sperm and the associated accessory gland fluid to the female during copulation.

## 1. Introduction

The blood-gorging hemipteran, *Rhodnius prolixus* Stål, 1859 (Hemiptera: Triatominae), is one of the model insects upon which the foundations of insect physiology have been laid [1,2,3,4]. *Rhodnius prolixus* is also medically important, being a principal vector of *Trypanosoma cruzi* (Chagas, 1909) (Kinetoplastida: Trypanosomatidae), the causative agent of Chagas disease, a neglected disease endemic to Central and South America [5]. Blood-gorging has a high epidemiological impact, initiating egg production in adult mated females, with each of several meals resulting in a batch of approximately 30–50 eggs, such that a female can produce hundreds of offspring [6]. Blood-gorging is also necessary for the adult males to initiate the development of sperm and the production of the accessory gland fluids. Although, based on the nutritional state, an unfed male can produce some living spermatozoa [6]. Understanding the reproductive biology of *R. prolixus* is, therefore, of some importance from a scientific perspective but also in a medical context.

The production of viable offspring is a complex task, involving courtship, mating, the making of eggs (oogenesis), ovulation, the fertilization of eggs and egg-laying (oviposition) [4,7,8,9]. The female must produce eggs at the appropriate time and deposit them in a suitable location after fertilization has occurred. Thus, the hormonal and neural control of these reproductive processes includes coordination via the central nervous system (CNS), midgut, fat body and reproductive tissues, allowing for a tightly synchronized and integrated behavior [4,8,9]. These have been well described in the adult female *R. prolixus* (reviewed by [4]) and indeed in *Locusta migratoria* (Linnaeus, 1758) (Orthoptera: Acrididae) [8,9,10,11] and *Drosophila melanogaster* Meigan, 1830 (Diptera: Drosophilidae) [12,13]. However, for successful egg production, the reproductive tract of the female must receive secretions, including sperm, from the male. Apart from receiving the sperm necessary for fertilization, however, the female is also changed behaviorally, physiologically and morphologically through mating. These changes are induced by the proteins and other molecules carried in the male’s ejaculate. These mating factors result in the mated females being quite different from virgin females [7,14,15,16,17,18,19]. The transfer of sperm and fluids occurs during copulation and is facilitated through the coordinated contractions of the muscles within the male reproductive system. In contrast to the female, less is known about the musculature or control of the contractions of the male reproductive system in insects, including *R. prolixus* [20,21].

**Figure 1:**
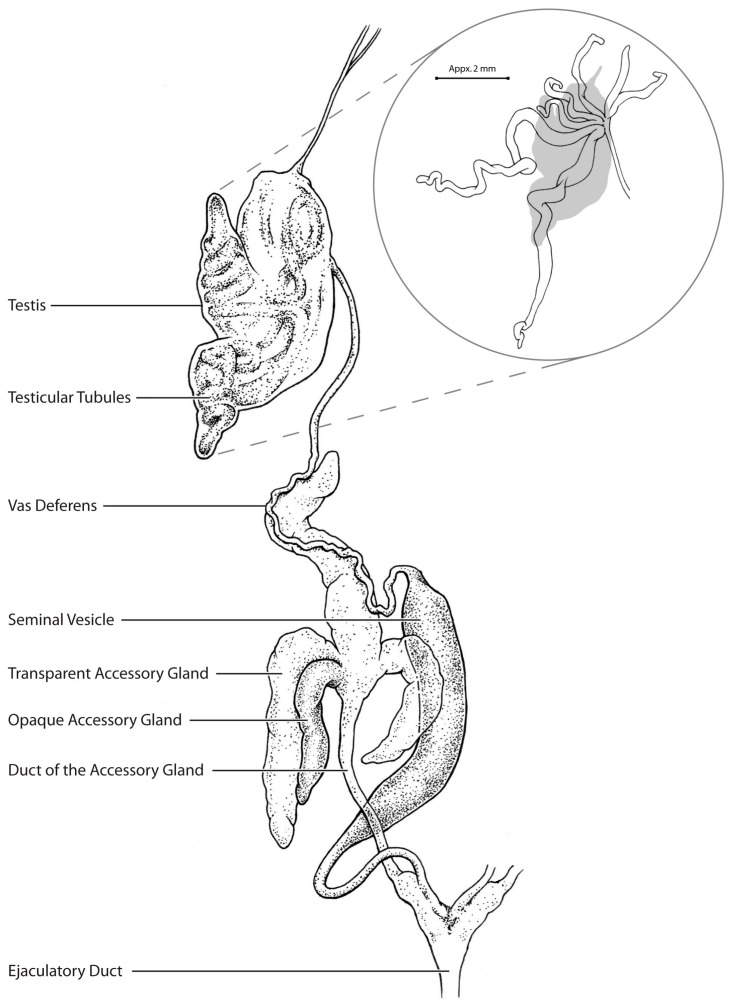
Diagrammatic representation of the left side of the male reproductive system of adult male *Rhodnius prolixus*. Note the vas deferens and seminal vesicle are pulled out from behind the accessory glands for clarity. The callout depicts the testis with the sheath removed and the seven follicles spread out to show their shape and length. Drawing by Zach McLaughlin.

The reproductive system of the male *R. prolixus* is bilaterally symmetrical with each side consisting of a testis, vas deferens, seminal vesicle and four accessory glands (three transparent and one opaque), with both sides joined together posteriorly via an ejaculatory duct (Figure 1; [22]). Each testis contains seven follicles bound together with a transparent connective tissue sheath [23], which are involved in sperm production. Spermatogenesis is cystic [24] and mature sperm from the testes are transported along the vas deferens and stored within the seminal vesicles. The accessory glands produce the proteinaceous material and a substance that is white and milky [19,25,26]. The spermatophore, containing sperm and the accessory gland secretions, is delivered to the female during copulation by way of contractions of the ejaculatory duct [17,22].

In parallel with the female reproductive system, the male reproductive system in *R. prolixus* is likely under neuroendocrine control, but also direct neural control via innervation from the branches of the mesothoracic ganglionic mass (MTGM) trunk nerves [27]. In addition, several studies have indicated the possible involvement of neuropeptides in the control of the muscular activity of the *R. prolixus* male reproductive system, with the nerve processes immunoreactive to some neuropeptide families traversing over the regions of the reproductive system, but also the expression of transcripts for the G protein-coupled receptors (GPCRs) in the reproductive system. In particular, the myoinhibiting peptide-like immunoreactive processes are associated with the male accessory glands [28] and the proctolin-like immunoreactive processes are present in all parts of the male reproductive system except the testes in *R. prolixus* [29]. In addition, the FMRFamide-like peptides (FLPs) and proctolin have been shown to be particularly relevant in the female reproductive system of insects, including *R. prolixus* [29,30,31,32,33,34,35].

Here we describe the muscle arrangements, the FMRFamide-like immunoreactivity (FLI), the expression of the GPCRs for the selected neuropeptides, as well as the effects of two extended FLPs, *R. prolixus* myosuppressin (RhoprMS) and proctolin on the muscle contraction in the male reproductive system of *R. prolixus*.

## 2. Materials and Methods

### 2.1. Animals

*Rhodnius prolixus* were reared in an incubator at 25 °C and 50% humidity at the University of Toronto Mississauga. The insects were fed on defibrinated rabbit blood through an artificial membrane (Cedarlane, Burlington, ON, Canada). All experiments were conducted on adult virgin males, approximately one week post-feeding as adults.

### 2.2. Chemicals

The *R. prolixus* neuropeptides, AKDNFIRFamide, GNDNFMRFamide and myosuppressin (pQDIDHVFMRFamide) were purchased from GenScript, Inc. (Piscataway, NJ, USA). Proctolin was purchased from Peninsula Laboratories, Inc. (Belmont, CA, USA). Stocks of 10^−3^ M were produced in double distilled water and stored as 40 µL aliquots at −20 °C. Physiological saline (150 mM NaCl, 8.6 mM KCl, 2 mM CaCl_2_, 4 mM NaHCO_3_, 34 mM glucose, 8.5 mM MgCl_2_ and 5 mM HEPES at pH 7.2) was prepared using double distilled water and used for immunohistochemistry and the dilution of the peptides for the contraction assays.

The rabbit anti-FMRFamide primary antibody as well as the Cy3 anti-rabbit, IgG, secondary antibody were purchased from the Jackson ImmunoResearch Laboratories, Inc. (West Grove, PA, USA) and the Alexa Fluor 594-conjugated goat anti-rabbit Ab was purchased from Life Technologies, Carlsbad, CA, USA). All antibodies were stored at −20 °C.

### 2.3. Phalloidin Staining

The phalloidin-tetramethylrhodamine B isothiocyanate conjugate (Sigma-Aldrich, Oakville, ON, Canada) was used as previously described [32] to stain F-actin in the muscle fibers. The male reproductive systems were fixed in 4% formaldehyde and diluted in a phosphate-buffered saline (PBS: 6.6 mM Na_2_HPO_4_/KH_2_PO_4_, 150 mM NaCl, pH 7.4) overnight at 4 °C. After three washes using PBS, the tissues were incubated with phalloidin-Cy3 (1:330 dilution in PBS) at room temperature for 45 min. The tissues were then washed in PBS and mounted on slides with one drop of Fluoroshield (Sigma-Aldrich, ON, Canada) to be viewed on a Zeiss laser scanning confocal microscope LSM 800 using the LSM image browser software (Carl Zeiss, Jena, Germany).

### 2.4. Whole Mount Immunohistochemistry

The male adult *R. prolixus* reproductive systems were dissected in physiological saline and fixed in 2% paraformaldehyde in Millonig’s buffer (130 mM NaH_2_PO_4_∙H_2_O, 100 mM NaOH, 1.2% glucose, 0.3 mM CaCl_2_∙2H_2_O, pH 7.0) overnight at 4 °C. After fixation and washing in PBS, staining was performed as previously described [32] using a rabbit anti-FMRFamide primary antiserum (1:1000 in PBS containing 0.4% Triton-X-100 and 2% normal goat serum (NGS)) for 48 h at 4 °C. After incubation, the tissues were washed in PBS and then incubated in either a goat anti-rabbit secondary antibody conjugated to Cy3 or Alexa-594 (1:600 in 10% NGS in PBS) for 24 h at 4 °C. The preparations were washed repeatedly in PBS and then either run through a glycerol series and mounted on glass slides in glycerol or mounted directly with Flouroshield (for DAPI). The slides were viewed through a Zeiss LSM 800 confocal laser microscope (Carl Zeiss, Jena, Germany). To control the specificity of the primary antiserum, the primary antiserum (1:1000) was pre-absorbed overnight at 4 °C with 10^−5^ M GNDNFMRFamide. This eliminated all immunoreactive staining, indicating that the staining was specific for the FLPs.

### 2.5. RNA Extraction and Quantitative Polymerase Chain Reaction (qPCR)

The adult male *R. prolixus* were dissected in cold autoclaved PBS (pH of 7.4). The reproductive tissues were then directly suspended in the TRIzol reagent (Invitrogen by Thermo Fisher Scientific, Waltham, MA, USA) and the total RNA was obtained according to the manufacturer’s instructions. The total yield of each sample was determined using a DS-11 Series-NanodropTM One spectrophotometer (DeNovix Inc., Wilmington, DE, USA). The total RNA extracts were then subjected to DNase treatment using the DNase I (RNase-free) Kit (Thermo Fisher Scientific, Mississauga, ON, Canada). The synthesis of the first-strand complementary DNA (cDNA) was conducted using the Applied Biosystems High Capacity cDNA Reverse Transcription Kit (Applied-Biosystems, by Thermo Fisher Scientific, Mississauga, ON, Canada). The conditions of the thermal cycler were 10 min at 25 °C, 120 min at 37 °C and 5 min at 85 °C. The cDNAs obtained were diluted 10-fold for the quantitative PCR (qPCR). The qPCR assays were performed using an advanced qPCR 1-Step Kit with Supergreen Dye Low-ROX (Wisent Bioproducts Inc., Saint-Jean-Baptiste, QC, Canada) according to manufacturer’s instructions and using a CFX384 TouchTM Real-Time PCR Detection System (Bio-Rad Laboratories, Inc., Mississauga, ON, Canada). The qPCR temperature cycling profile was an initial denaturation for 2 min at 95 °C followed by 40 cycles of 5 s at 94 °C, 30 s at 60 °C and the final melting curve from 65 °C to 95 °C in increments of 0.5 °C. *β-actin* and ribosomal protein 49 (*Rp49*) were used as the reference genes [36]. To assess the accuracy of the cDNA product amplification, the dissociation curves were examined and only a single peak produced for each pair of primers was observed. The primers are shown in Supplementary Table S1. The mRNA expression was calculated relative to 1000 copies of the geometric mean of the reference genes using the 2^−ΔCt^ method [37].

### 2.6. Contraction Assays

The effect of the peptide application on the frequency of the contractions of the transparent accessory gland, seminal vesicle and ejaculatory duct of the male adult *R. prolixus* were investigated for proctolin, the extended FLPs, AKDNFIRFamide and GNDNFMRFamide and myosuppressin. The insects were dissected by removing the legs and wings and then removing the dorsal cuticle, the underlying abdominal fat body and the digestive system. All innervation was severed from the CNS to the reproductive system. This dissection allowed the reproductive system to remain within the body of the insect, which could be filled with physiological saline and used as a bath containing 200 µL of saline. Two electrodes fashioned from minuten pins connected to an impedance convertor (UFI model 2991, Morro Bay, CA, USA) were placed on either side of the contracting tissue and the contractions were recorded and analyzed with the use of the LabChart software (ADI Instruments, Colorado Springs, CO, USA). The contractions were monitored before, during and following the application of the peptides. The impedance converter was set to the AC mode for the quantification of the frequency of the contractions.

All preparations were maintained and monitored in the bath of 200 μL of the physiological saline. The saline (control) or the peptides in saline were applied to the preparation by simultaneously adding 100 μL of saline or saline containing two times the final concentration of the peptide, while removing 100 μL of the saline from the bath. The preparations were monitored for 1 min and then washed with saline. The dose response curves compared the frequency of the contractions after the peptide application to the frequency in the saline prior to the addition of the peptide. For examining the inhibitory action of myosuppressin on the frequency of the contraction, myosuppressin at varying concentrations was applied to a preparation that had been stimulated by a standard dose of proctolin (5 × 10^−9^ M) which gave a robust response. A dose response comparing the frequency after the myosuppressin addition to that of the standard dose of proctolin was used to examine the minimum and maximum effect of the myosuppressin.

### 2.7. Statistical Analyses

Graph Pad Prism 9 (GraphPad Software, San Diego, CA, USA) was used to conduct the statistical analyses and to construct the graphical representations used in this study. The one-way ANOVA followed by the post-hoc Tukey’s test was used in order to determine the statistical significance. A significance of *p* < 0.05 was used for the statistical significance.

## 3. Results

**Figure 2:**
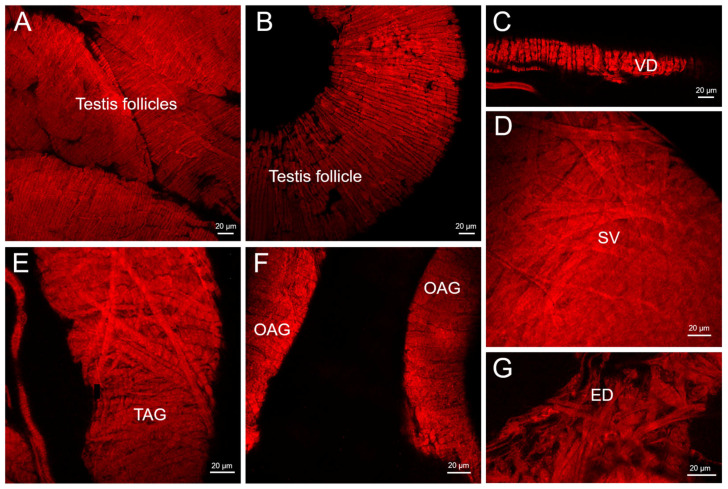
Phalloidin staining of muscle F-actin in the reproductive system of male adult *R. prolixus*. (**A**,**B**) The muscle fiber network encircling each follicle of the testis, (**C**) a circular layer of muscle fiber surrounds the vas deferens (VD), (**D**) the seminal vesicle (SV) has a crisscross pattern of large diameter muscle fibers, (**E**) transparent accessory gland (TAG) with large diameter muscle fibers in a crisscross and circular pattern, (**F**) the opaque accessory gland (OAG) with a circular layer of muscle fibers and (**G**) the ejaculatory duct (ED) has large diameter muscle fibers arranged in a crisscross pattern. Representative images from 10 preparations.

All the structures of the male reproductive system (Figure 1) were covered in layers of muscle fibers, as shown by the phalloidin staining of F-actin (Figure 2). The testis was an ovoid structure lined by a collagenous membrane that enclosed the seven testicular follicles, two of which were longer than the others (Figure 1). The follicles of the testis were surrounded by slender muscle fibers (1.5–3 µm diameter for the smaller follicles and 4–6 µm in diameter for the larger follicles) that encircled each of the follicles (Figure 2A,B). Similarly, the muscle fibers (3–5 µm in diameter) were arranged in a circular pattern surrounding the vas deferens (Figure 2C). The muscle layers of the seminal vesicle formed a crisscross pattern of larger diameter muscle fibers (7 µm in diameter) (Figure 2D) as was also evident for the transparent accessory glands (Figure 2E). The opaque accessory glands had muscle layers that more resembled those seen in the follicles, i.e., in a circular pattern (Figure 2F), whereas the ejaculatory duct had large diameter muscle fibers (7 µm in diameter; Figure 2G) that created a dense and complex crisscross pattern.

**Figure 3:**
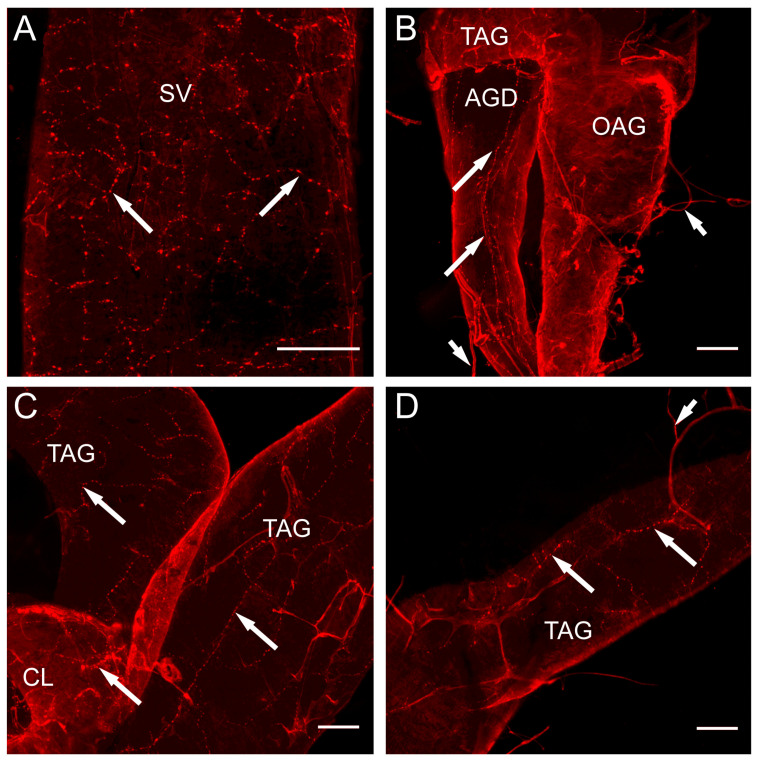
FMRFamide-like immunoreactivity (FLI) is associated with the male reproductive system in *R. prolixus*. (**A**) A network of FMRFamide-like immunoreactive processes and blebs (long arrows) are present on the seminal vesicle (SV). (**B**) FMRFamide-like immunoreactive processes and blebs (long arrows), coming from the nerves (short arrows) that branch off the trunk nerve, run longitudinally along the accessory gland duct (AGD). Minimal FLI is seen on the opaque accessory gland (OAG). (**C**,**D**) FLI seen in processes and blebs (long arrows) branching over the transparent accessory glands (TAG) and calyx (CL). Note the nerve (short arrow) with FMRFamide-like immunoreactive processes arriving at the TAG. Scale bar represents 100 µm. Representative images from 20 preparations.

The nerve processes displaying the FLI were found distributed over all the structures of the male reproductive system except for the testis (Figure 3). Numerous immunoreactive processes and blebs were found over the entire seminal vesicle (Figure 3A). Figure 3B–D illustrate the nerves immunoreactive for the FLPs arising from the trunk nerves (abdominal nerve V, AbN V) of the MTGM [27] that projected the axonal processes along the duct of the accessory glands (Figure 3B) and onto the transparent and opaque accessory glands, where they produced fine branching processes and blebs (Figure 3C,D).

**Figure 4:**
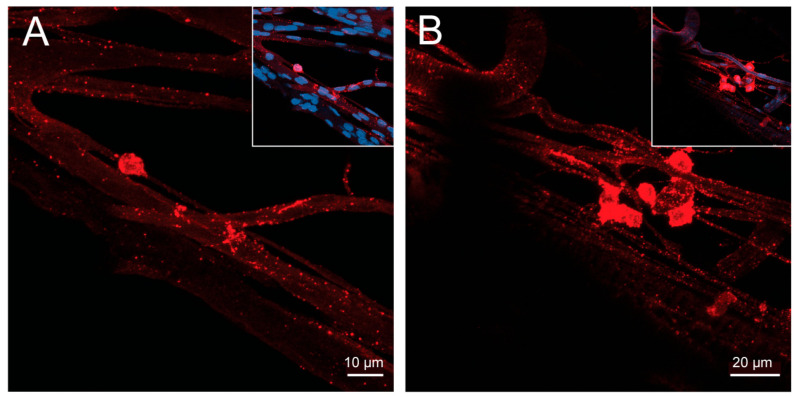
FMRFamide-like immunoreactivity (FLI) is associated with peripheral neurosecretory neurons laying on the nerves. (**A**) shows a peripheral neurosecretory neuron, and (**B**) shows a group of peripheral neurosecretory neurons with associated neurohemal areas on small nerves branching from the trunk nerve lying between the accessory glands. Insets show the nuclei of neurons also stained with DAPI (blue). Representative images from four preparations.

Interestingly, several FMRFamide-like immunoreactive neurosecretory cell bodies and their associated neurohemal sites were found on the nerves projecting around and onto the accessory gland and the seminal vesicle (Figure 4A,B).

**Figure 5:**
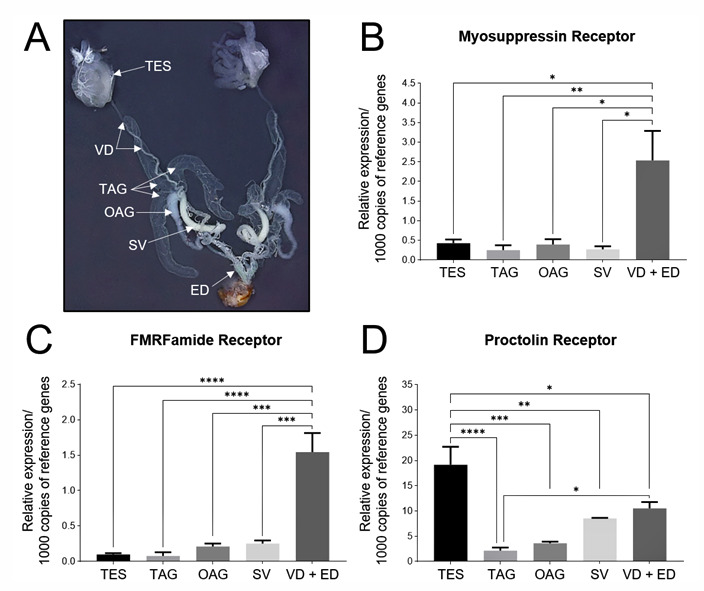
Transcripts for the neuropeptide receptors are expressed in the male reproductive system in *R. prolixus*. (**A**) Photograph of the male reproductive system with one testis (TES) de-sheathed to show the follicles, the vas deferens (VD), transparent accessory glands (TAG), opaque accessory gland (OAG), seminal vesicle (SV) and ejaculatory duct (ED). The relative transcript expression of (**B**) the *myosuppressin receptor*, (**C**) the extended *FMRFamide receptor* and (**D**) the *proctolin receptor* in the various structures of the male reproductive system. The y-axes represent the relative expression obtained via geometric averaging using *Rp49* and *β-actin* as the reference genes. The results are shown as the mean ± SEM (*n* = 4, where *n* represents a pool of tissue from three insects). The statistical significance of the data was measured using a one-way ANOVA test and Tukey’s post-hoc multiple comparison test. A *p* value of <0.05 was considered statistically significant and is denoted using asterisks above the bars. * *p* < 0.05, ** *p* < 0.01, *** *p* < 0.001 and **** *p* < 0.0001.

Since the FLI was present in the innervation to the male reproductive system and previously we have shown that a proctolin-like immunoreactivity was also present [29], we analyzed the various structures to determine if the transcripts for the receptors for these families were also present (Figure 5). Two families of FLPs were first examined: the myosuppressin and the extended FMRFamides [31]. The transcripts for both of their receptors were expressed in all the structures of the male reproductive tissue with the highest amount found in the vas deferens and the ejaculatory duct (Figure 5B,C). In contrast, whilst the *proctolin receptor* transcript was also found in all the structures of the male reproductive system, the highest amount was found in the testis, followed by the vas deferens and ejaculatory duct (Figure 5D).

**Figure 6:**
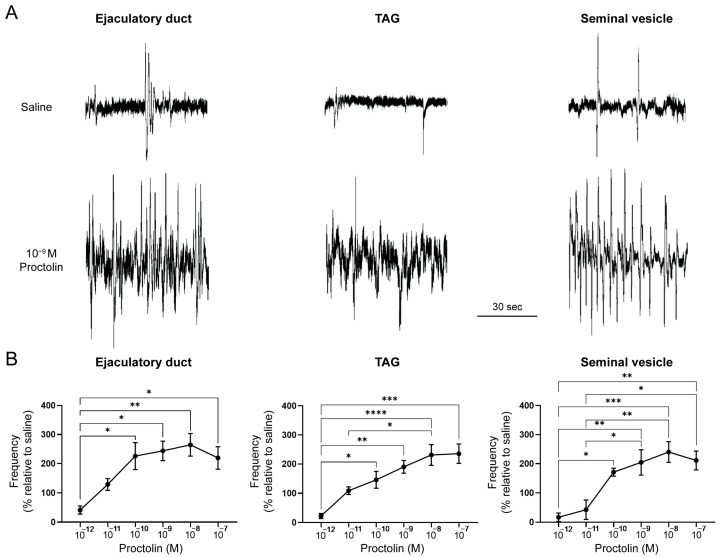
The ejaculatory duct, transparent accessory glands and seminal vesicle display spontaneous contractions, which can be stimulated by proctolin. (**A**) Spontaneous contractions of the ejaculatory duct, transparent accessory gland (TAG) and seminal vesicle can be simulated by proctolin (10^−9^ M). Representative traces from six–eight preparations. (**B**) Proctolin leads to a dose-dependent increase in the frequency of muscle contractions in the ejaculatory duct, the transparent accessory gland (TAG) and the seminal vesicle. The results are shown as the mean ± SEM (*n* = 4–6). The statistical significance of the data was measured using a one-way ANOVA test and Tukey’s post-hoc multiple comparison test. A *p* value of <0.05 was considered statistically significant and is denoted using asterisks above the bars. * *p* < 0.05, ** *p* < 0.01, *** *p* < 0.001 and **** *p* < 0.0001.

The seminal vesicle, the transparent accessory glands and the ejaculatory duct in the adult male *R. prolixus* were myogenic and individually exhibited spontaneous contractions in vitro at a frequency of 5.8 ± 0.6, 4.9 ± 0.3 and 5.7 ± 0.3 contractions per minute, respectively (Figure 6A), with some contractions being small and others larger. The testes and opaque accessory glands showed little or no muscular activity (data not shown). The application of proctolin to the ejaculatory duct, transparent accessory gland or the seminal vesicle resulted in similar dose-dependent increases in the number of contractions with a threshold of approximately 10^−12^ M and a maximum around 10^−8^ M (Figure 6B). The maximum response resulted in an increase in the frequency of contractions by approximately 250% for each tissue (Figure 6).

**Figure 7:**
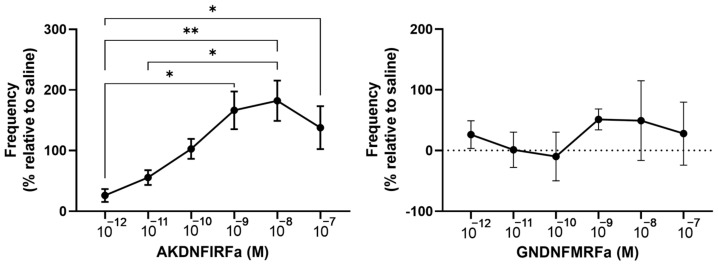
The effects of two extended FMRFamides, AKDNFIRFamide and GNDNFMRFamide, on the contraction frequency of the ejaculatory duct of the male reproductive system. AKDNFIRFamide led to a dose-dependent increase in the frequency of the muscle contractions in the ejaculatory duct, whereas GNDNFMRFamide did not alter the frequency. The results are shown as the mean ± SEM (*n* = 4–5). The statistical significance of the data was measured using a one-way ANOVA test and Tukey’s post-hoc multiple comparison test. A *p* value of <0.05 was considered statistically significant and is denoted using asterisks above the bars. * *p* < 0.05, ** *p* < 0.01.

Within the *R. prolixus* neuropeptidome, the extended FMRFamide family was composed of peptides that contained two different C-terminal endings: FMRFamide and FIRFamide [38,39]. Here, AKDNFIRFamide and GNDNFMRFamide were tested in the contraction assays on the ejaculatory duct since this was the most robust preparation. A similar dose-dependent effect to that seen for proctolin was found for AKDNFIRFamide on the ejaculatory duct with a maximum increase of 182.1 ± 33.3% at 10^−8^ M (Figure 7). Interestingly, GNDNFMRFamide did not result in any statistical change in the contraction frequency (Figure 7).

**Figure 8:**
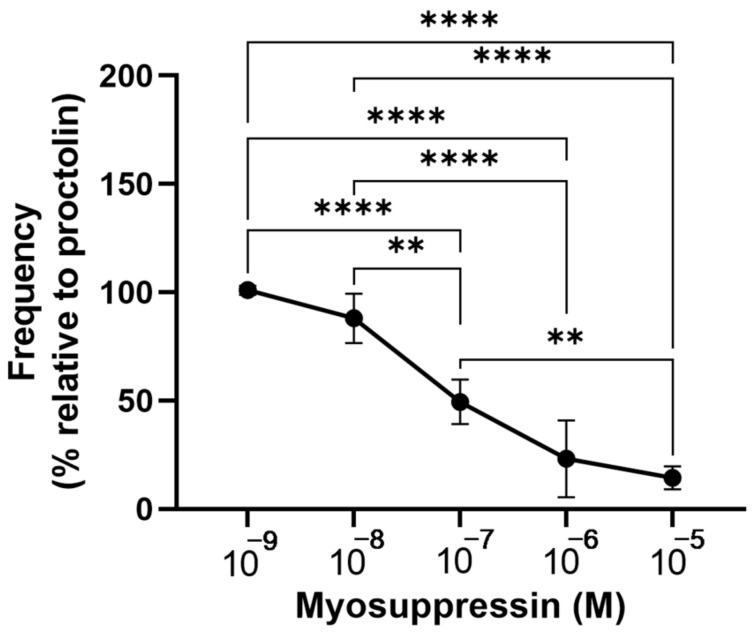
Myosuppressin inhibits a proctolin-induced increase in the frequency of the contractions of the ejaculatory duct in the male reproductive system. Myosuppressin dose-dependently inhibits the increase in the frequency of contractions induced by proctolin (5 × 10^−9^ M). The results are shown as the mean ± SEM (*n* = 4). The statistical significance of the data was measured by a one-way ANOVA test and Tukey’s post-hoc multiple comparison test. A *p* value of <0.05 was considered statistically significant and is denoted using asterisks above the bars. ** *p* < 0.01 and **** *p* < 0.0001.

The myosuppressin in *R. prolixus,* RhoprMS, had a unique amino acid sequence for the insects of pQDIDHVFMRFamide [40], but RhoprMS was still capable of inhibiting a proctolin-induced increase in the contraction frequency of the ejaculatory duct in a dose-dependent manner (Figure 8) with a threshold between 10^−9^ M and 10^−8^ M and a maximum of 88% inhibition at 10^−5^ M.

## 4. Discussion

The FLPs are a large family of structurally similar neuropeptides with diverse biological activities [31,41]. There are many subfamilies present within this family, one of which is referred to as the extended FLPs and another as the myosuppressins [41]. Insect myosuppressins are a family of FLPs with a relatively conserved sequence of X_1_DVX_4_HX_6_FLRFamide (where X_1_ = pQ, P, T or A, X_4_ = D, G, or V and X_6_ = V or S), although RhoprMS has a unique peptide sequence consisting of pQDIDHVFMRFamide [40]. Despite these differences, RhoprMS is still functionally a myoinhibitory peptide, decreasing the heart rate and inhibiting the anterior midgut and hindgut contractions in *R.prolixus* [40]. In addition, a functional receptor expression assay confirmed that the cloned RhoprMS receptor was indeed activated by RhoprMS [42]. The FLPs were localized in the cell bodies and processed throughout the CNS, and in processes on the peripheral tissues of many insects, including *R. prolixus* (see [31,32]). Moreover, extensive work was carried out on the effects of the FLPs on the physiology of the insect female reproductive system [31,32,43], but little is known about the role of the FLPs on male reproduction in *R. prolixus*. Similarly, proctolin, the first insect neuropeptide to be sequenced and synthesized [44], was present within the neurons that project the skeletal and visceral muscle (including female and male reproductive structures), where it stimulated or potentiated muscle contractions (see [30,45,46,47,48]). Interestingly, unlike most neuropeptides which exist as members of extended families, proctolin was almost invariably present in one form, that of the pentapeptide RYLPT [48]. The sequencing of the *R. prolixus* genome [49] and its neuropeptidome [38,39] revealed the sequences of a variety of *R. prolixus* FLPs, which, along with proctolin, may now be tested on their presence and ability to alter contractions of the male reproductive system.

The male reproductive system of *R. prolixus* is composed of muscular tissues each performing contractions that aid in the transfer of sperm and fluid into the female during mating [4,7]. Most parts of the male reproductive system, namely the seminal vesicles, transparent accessory glands and the ejaculatory duct, exhibit a spontaneous contractile activity of various frequencies and characteristics that must be coordinated for the successful transfer of sperm and proteinaceous secretions. The testes, which produce the sperm, were each composed of seven follicles that are held together by a membranous sheath, but little or no contractions were evident, although the follicles themselves might have spontaneously but weakly contracted. Similarly, little or no spontaneous contractions were seen in the opaque accessory glands, although our impedance method was restricted to the frequency and not the force. Interestingly, the various patterns of the muscle fiber layers from thin circular to more complex crisscross patterns implied subtle differences in the form of the contractions and movement of each of the structures, allowing for waves of contractions or twisting patterns. These contractions presumably aided in the movement of sperm out of the testis, into the vas deferens, seminal vesicle and then the ejaculatory duct, combined with the proteins and lipids from the transparent and opaque accessory glands. The sperm and associated fluids form a spermatophore, which is ejaculated into the female during copulation [7]. The various muscle fiber diameters and pattern layers may be related to the viscosity and volume of the fluid that is being moved. Thus, the slender muscle fibers on the testicular follicles, which produce sperm, and the vas deferens, which transport sperm for storage to the seminal vesicles, are sufficient to move the sperm into the seminal vesicles, but the seminal vesicles require larger diameter fibers to eject the more viscous fluid into the ejaculatory duct. Similarly, the white fluid in the opaque accessory glands may be more viscous than the fluid in the transparent accessory glands, accounting for their thicker muscle fibers. The thicker muscle fibers in the accessory gland duct and ejaculatory duct are probably needed to eject the combined sperm and fluids through the narrow ducts into the female [50].

The distribution of the FLI throughout the CNS and reproductive tissues of the female adult *R. prolixus* was described [32]. The antibody recognized all FLPs ending in the RFamide sequence and was not specific for any subfamily. In the male reproductive system, the FLI was present in the nerves innervating all the structures of the male reproductive system except for the testis. The peripheral neurosecretory cells with the FLI were also present indicating an additional more local neurohormonal control. Interestingly, in the cockroach *Leucophaea maderae*, peripheral neurons were found within a fine network of nerve fibers covering the entire surface of the male accessory glands [51]. A previous study on *R. prolixus* also reported the presence of proctolin-like immunoreactive processes over all parts of the male reproductive system except the testis [29]. These observations, coupled with the transcript expression for the proctolin receptor, the extended FMRFamide receptor and the myosuppressin receptor, would imply that these neuropeptides are involved in the coordination of the contractions of the various parts of the male reproductive system. This was confirmed using in vitro contraction assays whereby proctolin and AKDNFIRFamide were each capable of dose-dependently increasing the frequency of contractions of the ejaculatory duct, transparent accessory gland and the seminal vesicle. Interestingly, GNDNFMRFamide did not alter the contractions, indicating the importance of the N-terminal amino acid sequence for binding to the receptor. A similar observation was seen in the female *R. prolixus* reproductive tissues, where GNDNFMRFamide was less effective than AKDNFIRFamide on stimulating the contractions in the ovaries, oviduct and bursa [32]. The myosuppressin inhibited the proctolin-induced contractions of the ejaculatory duct. Thus, these neuropeptides can be used to fine tune contractions such that the successful deposition of the sperm and fluids can occur during mating. Similar conclusions have been drawn in some other insects, including *Euborellia annulipes* (Lucas 1847) (Dermaptera: Anisolabididae), *Tenebrio molitor* Linnaeus, 1758 (Coleoptera: Tenebrionidae) and *Zophabus atratus* (Fabricius, 1775) [33,34,35,52]. The stimulatory and inhibitory control over the muscle contractions was shown in the various parts of the female *R. prolixus* reproductive system [4,30,32], as it has been for other insects, particularly *D. melanogaster* (see [12,13]). Thus, there are times when the muscles must be stimulated to contract and other times when relaxation is required. Indeed, the control of the visceral muscle associated with reproductive structures can be quite complicated, including the involvement of biogenic amines and neuropeptides, and also the co-localization of these neuroactive chemicals with more classical neurotransmitters such as glutamate [10,11,13,46].

Overall, we described the gross anatomy of the male reproductive structures in *R. prolixus* and their associated musculature. Proctolin was a powerful stimulator of male contractions as it is on female reproductive structures (see [47,48]), including those of *R. prolixus* [4,30]. Immunohistochemistry also showed that the FLPs were associated with the neural processes on the muscle fibers of nearly all the reproductive structures in the male *R. prolixus* and in the associated peripheral neurosecretory cells, and they most likely play roles in the control of their contractile rhythms via both direct neural innervation or as a neurohormone. The various regions of the male reproductive system express transcripts for the receptors of proctolin, myosuppressin and the extended FMRFamides, supporting the contraction assays that show proctolin and AKDNFIRFamide to be strong stimulators of the contractions with RhoprMS acting as an inhibitor of the contractions. Taken all together, this work implicates these families of peptides in coordinating the male reproductive structures for a successful transfer of sperm and associated accessory gland fluids.

## Data Availability

The data that supports the findings of this study are available in the manuscript and/or available upon request from the authors.

## Supplementary Materials

The following supporting information can be downloaded at: https://www.mdpi.com/article/10.3390/insects14040324/s1, Table S1: Primers used for qPCR.
