# Supplementary material for: The Male Reproductive System of the Kissing Bug, Rhodnius prolixus Stål, 1859 (Hemiptera: Reduviidae: Triatominae): Arrangements of the Muscles and the Myoactivity of the Selected Neuropeptides
Source: Insects. 2023 Mar 28;14(4):324. doi: 10.3390/insects14040324 (PMC10146185; doi:10.3390/insects14040324)
Supplement: Supplementary file 1 [file insects-14-00324-s001.zip › insects-2299353-supplementary.pdf]

Supporting Information for

The Male Reproductive System of the Kissing Bug, *Rhodnius prolixus* Stål, 1859 (Hemiptera: Reduviidae: Triatominae): Arrangements of the Muscles and the Myoactivity of the Selected Neuropeptides

Angela B. Lange *, Anika Kisana, Jimena Leyria and Ian Orchard

* Correspondence: angela.lange@utoronto.ca

Supplementary Table S1. Primers used for qPCR.

Gene code	Primers to qPCR	Sequence (5→3)
KC004226.1	Myosuppressin R_forward	CTCTGTGGCCAATCTGCTCA
	Myosuppressin R_reverse	TCCATCCGTAGGTGTAGCGA
RPRC001551	FMRFamide R_forward	ATGTGATGGTGGCGTTAGGG
	FMRFamide R_reverse	GCTCTGGCTGCTCGTTTTAC
RPRC015267	Proctolin R_forward	TGGTTGGGGCAACATCCTAC
	Proctolin R_reverse	TTCGTACTGCAGCCACTAGC
RPRC009875	Actin_forward	AGAGAAAAGATGACGCAGATAATGT
	Actin _reverse	ATATCCCTAACAATTTCACGTTCG
RPRC014419	Rp49_forward	GTGAAACTCAGGAGAAATTGGC
	Rp49_reverse	AGGACACACCATGCGCTATC
